# Supplementary material for: Predicting visual function by interpreting a neuronal wiring diagram
Source: Nature. 2024 Oct 2;634(8032):113–23. doi: 10.1038/s41586-024-07953-5 (PMC11446822; doi:10.1038/s41586-024-07953-5)
Supplement: Supplementary file 6 — CRF and ERF predictions for individual TmY4 and TmY9 cells. Analogous to Supplementary Data 3, but for TmY target types. Shown are the top four monosynaptic pathways, the strongest pathway passing through each of the top ten intermediary types (ranking from Extended Data Fig. 7), and the trisynaptic pathway Tm1–TmY–Dm3–TmY (see the section entitled Prediction of spatial normalization). [file 41586_2024_7953_MOESM6_ESM.zip › DataS4/TmY9q/720575940631441711.pdf]

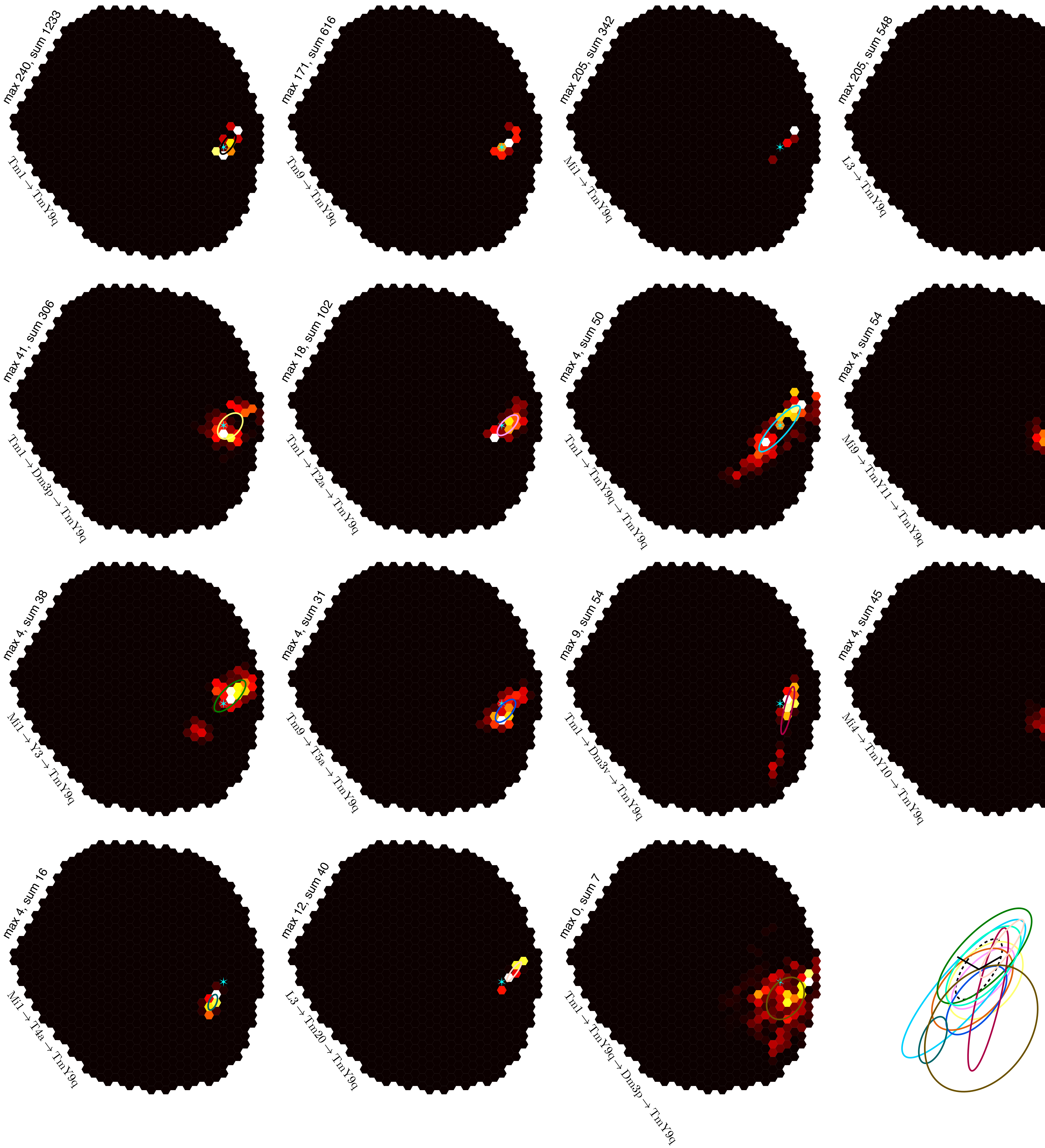
max 240, sum 1233
Tm1 → TmY9q
max 171, sum 616
Tm9 → TmY9q
max 205, sum 342
Mi1 → TmY9q
max 205, sum 548
L3 → TmY9q
max 41, sum 306
Tm1 → Dm3p → TmY9q
max 18, sum 102
Tm1 → T2a → TmY9q
max 4, sum 50
Tm1 → TmY9q → TmY9q
max 4, sum 54
Mi9 → TmY11 → TmY9q
max 4, sum 38
Mi1 → Y3 → TmY9q
max 4, sum 31
Tm9 → T5a → TmY9q
max 9, sum 54
Tm1 → Dm3v → TmY9q
max 4, sum 45
Mi4 → TmY10 → TmY9q
max 4, sum 16
Mi1 → T4a → TmY9q
max 12, sum 40
L3 → Tm20 → TmY9q
max 0, sum 7
Tm1 → TmY9q → Dm3p → TmY9q
